# Evaluating rehabilitation following lumbar fusion surgery (REFS): study protocol for a randomised controlled trial

**DOI:** 10.1186/s13063-015-0751-9

**Published:** 2015-06-04

**Authors:** James Greenwood, Alison McGregor, Fiona Jones, Michael Hurley

**Affiliations:** Internal Box 8, Victor Horsely Department of Neurosurgery, National Hospital of Neurology and Neurosurgery, Queen Square, London, WC1 3BG UK; Biodynamics Lab, Imperial College London, Charing Cross Hospital, Charing Cross Campus, London, W6 8RP UK; St Georges University of London, Faculty of Health and Social Care Sciences, 2nd Floor Grosvenor Wing, Cranmer Terrace, London, SW17 0RE UK

**Keywords:** Lumbar fusion, Rehabilitation, Physiotherapy, Qualitative methodology, Post-operative exercise

## Abstract

**Background:**

The rate of lumbar fusion surgery (LFS) is increasing. Clinical recovery often lags technical outcome. Approximately 40 % of patients undergoing LFS rate themselves as symptomatically unchanged or worse following surgery. There is little research describing rehabilitation following LFS with no clear consensus as to what constitutes the optimum strategy. It is important to develop appropriate rehabilitation strategies to help patients manage pain and recover lost function following LFS.

**Methods/design:**

The study design is a randomised controlled feasibility trial exploring the feasibility of providing a complex multi-method rehabilitation intervention 3 months following LFS. The rehabilitation protocol that we have developed involves small participant groups of therapist led structured education utilising principles of cognitive behavioral therapy (CBT), progressive, individualised exercise and peer support. Participants will be randomly allocated to either usual care (UC) or the rehabilitation group (RG). We will recruit 50 subjects, planning to undergo LFS, over 30 months. Following LFS all participants will experience normal care for the first 3 months. Subsequent to a satisfactory 3 month surgical review they will commence their allocated post-operative treatment (RG or UC). Data collection will occur at baseline (pre-operatively), 3, 6 and 12 months post-operatively. Primary outcomes will include an assessment of feasibility factors (including recruitment and compliance). Secondary outcomes will evaluate the acceptability and characteristics of a limited cluster of quantitative measures including the Oswestry Disability Index (ODI) and an aggregated assessment of physical function (walking 50 yards, ascend/descend a flight of stairs). A nested qualitative study will evaluate participants’ experiences.

**Discussion:**

This study will evaluate the feasibility of providing complex, structured rehabilitation in small groups 3 months following technically successful LFS. We will identify strengths and weakness of the proposed protocol and the usefulness and characteristics of the planned outcome measures. This will help shape the development of rehabilitation strategies and inform future work aimed at evaluating clinical efficacy.

**Trial registration:**

ISRCTN60891364, 10/07/2014.

**Electronic supplementary material:**

The online version of this article (doi:10.1186/s13063-015-0751-9) contains supplementary material, which is available to authorized users.

## Background

Instrumented lumbar fusion surgery (LFS) is undertaken to rigidly stabilise adjacent vertebral motion segments, commonly performed simultaneously with decompression of affected neural tissue, to relieve back and/or neurogenic leg pain [1–3]. Common clinical indications include symptomatic disc disease, segmental instability, and spinal stenosis [4–6]. The rate of LFS is increasing in the UK with over 6,547 fusions performed in 2012/13 [7]. A similar trend of escalating LFS rates has been reported in the US [8].

Following instrumented LFS 15 % of patients show no improvement and as many as 40 % of patients are unsure/dissatisfied with the outcome 2 years postoperatively [9–12], reporting ongoing back pain and related limitation in daily function [13, 14]. Similar findings have been reported in surgery for lumbar spine stenosis where functional recovery lags behind surgical outcome, [15]. This adds to the already considerable burden of years lived with disability (YLD) as a consequence of low back pain (LBP) [16].

The financial implications are also significant. The direct costs of LFS utilising titanium cages is reported to be between £9,000 to £11,000 per case [9, 17]. In the US the cost per quality-adjusted life year (QUALY) has been estimated at £25,000 [18]. Overall costs at 2 years (direct and indirect), including lost productivity, are significantly higher at £78,000 [19]. In the UK National Health Service (NHS), surgery represents the greatest single expenditure in the management of chronic low back pain (CLBP) [6].

A recent report by the Cochrane Back Review Group (CBRG), concluded that active rehabilitation was more effective than usual care (UC) with respect to functional recovery following laminectomy for lumbar stenosis [20]. This suggests that rehabilitation can facilitate recovery from some forms of spinal surgery.

Few published studies have looked at rehabilitation following LFS. Christensen *et al.*, [21] showed rehabilitation involving directed exercise and a ‘back Café’ (peer support group) improved pain and function compared to UC. Similar results were reported by Abbot *et al.* [22], in which psychomotor therapy (home exercises and outpatient appointments targeting maladaptive pain cognition, behaviour and motor control exercises) significantly reduced disability and pain compared with a physical rehabilitation regime. Rehabilitation in this study [22] began immediately following surgery, which is not standard practice in many units. The issue of timing may be relevant, as rehabilitation commenced 6 weeks postoperatively has been associated with inferior outcomes when compared to that commenced at 3 months [23].

A recent systematic review reported inconclusive, very low quality evidence for the effectiveness of physiotherapy management following LFS [24]. This is largely due to the lack of good-quality studies, the authors identifying the two studies summarised above, as eligible for inclusion. However, both of these studies reported a positive effect in favour of complex rehabilitation over physical exercise [22] or UC [21]. This paucity in the literature represents a gap in our understanding as to what constitutes best practice for rehabilitation following LFS, echoed by others [25, 26]. Therefore, it is vital to develop strategies to improve outcomes both in terms of human function and cost following this operation.

### Aims and objectives

The aim of this study is to evaluate the feasibility of providing complex rehabilitation in a group setting 3 months after technically successful instrumented LFS. This will include an analysis of our recruitment strategy, compliance with the study protocol and rehabilitation intervention. It will also evaluate the acceptability and characteristics (including population mean, SD and effect size) of a cluster of quantitative outcome measures. This will help achieve a more detailed analysis of the patient group under evaluation and thus a robust basis for the analyses of future work evaluating clinical efficacy. The nested qualitative analysis will identify strengths and weakness of the proposed protocol. This study will provide useful data for the development, refinement, and analysis of complex rehabilitation following instrumented LFS.

## Methods

### Design of trial

Rehabilitation following fusion surgery (REFS) is a randomised, controlled, multi-method, single-centre, feasibility trial.

### Participants (inclusion and exclusion criteria)

**Fig. 1:**
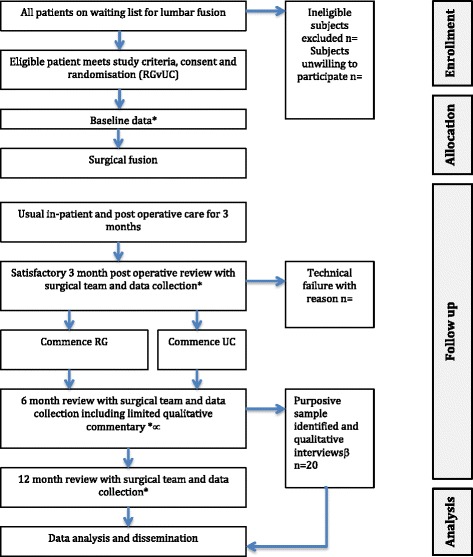
Trial Flowchart. ^*^Quantitative data collection, Oswestry disability index (ODI), pain self-efficacy questionnaire (PSEQ), aggregated functional performance test (AFPT), hospital anxiety and depression score (HADS), Client Services Receipt Inventory (CSRI), (European quality of life questionnaire-5 dimensions (EQ-5D); ^∝^qualitative commentary data collection; ^β^detailed qualitative interviews data collection. RG, rehabilitation group; UC, usual care

A convenience sample of subjects (n = 50) will be recruited from the Neurosurgical Department of the National Hospital for Neurology and Neurosurgery, (NHNN, UCLH, NHS Foundation Trust). The Complex Spine Team at NHNN performs approximately 60 fusion procedures per annum. If 33 % of these patients are eligible and consent, we will meet our recruitment target in 30 months. A local exploratory study suggested this recruitment strategy is achievable; contingency plans are in place should recruitment prove inadequate. A study overview is shown in Fig. 1.

Patients 18 to 75 years old will be included. Patients will be excluded if they have spinal cord involvement; have postoperative complications (infection, loosening or other technical failure of the surgical site that in the opinion of the attending surgeon precludes participation in rehabilitation); have had revision LFS (previous history of discectomy/decompression surgery are eligible); have lower limb joint pain that interferes with assessment or the ability to exercise; are unable to walk further than 20 m; have severe, poorly controlled psychological or physical comorbidity; have inadequate verbal and written English, or are unable/unwilling to undertake exercise, attend the postoperative programme or give signed consent.

### Recruitment/consent

Potential participants will be identified from the neurosurgical waiting list by JG. All patients undergoing LFS undergo a pre-operative assessment (PAS) to establish fitness for surgery. When patients are contacted to arrange the PAS the Chief Investigator (CI) will raise the possibility of the study. Those agreeing to consider participation will be sent a patient information sheet (PIS) (Additional file 1). At the PAS the CI will discuss any aspects of the study that are unclear. Patients who agree to participate will be asked to provide written informed consent (Additional file 2) and baseline data (BLD). These will be recorded, the participant’s general practitioner (GP) will be informed and they will be randomised to either RG or UC by block randomisation.

### Ethics/governance

The study received favorable ethical approval from the local Research Ethics Committee (REC), Queen Square, number 14/LO/0748. Sponsorship is provided by the R and D department at UCLH NHS Foundation Trust. A trial steering group consisting of the CI, academic supervisors, expert patients, statistician, expert in qualitative methodologies, consultant neurosurgeon and the interventional physiotherapists will meet bi-annually. Annual reporting of trial progress will be submitted to the REC and the funding body (National Institute for Health Research, NIHR). Adverse events will be reported to the CI for action in accordance with the defined stopping rules.

### Surgical procedures

All surgery will be performed by a member of the Complex Spine Team at NHNN. The surgical approach will be entirely at the discretion of the attending surgeon. In all cases however, this will involve rigid instrumentation, with or without surgical decompression of the relevant nerve roots/central canal. Records will be kept for reporting of the exact surgical procedure utilised.

### Randomisation and blinding

It is not possible to blind subjects to their allocation. Randomisation to either RG or UC will be by block randomization, utilising codes generated independently by the trial statistician at the Faculty of Health and Social Care Sciences, St Georges University of London (HEI). Concealment of allocation will be achieved by the remote generation of codes and the use of sealed opaque envelopes, numbered sequentially.

### Immediate postoperative care

Following LFS both groups will remain in hospital for approximately 5 days, experiencing identical care, including early ambulation, (usually within 6 h of the operation), check radiographs, wound monitoring, pain control, routine nursing observations, physiotherapy mobility check/advice and antithrombotic exercises.

The standard advice given to all patients following LFS in our unit is to avoid heavy lifting (nothing more than a kettle) for 3 months. This allows wound healing and encourages consolidation of the osseous fusion. During this period patients are encouraged to gradually increase their outdoor mobility with short regular walks to a maximum of 2 miles/day. All subjects will follow this standard advice for the first 3 months following surgery, irrespective of group allocation. A check appointment 6 weeks postoperatively with the surgical team monitors recovery.

At 3 months following surgery, if the surgical team is satisfied with the technical aspects of the surgery (no indication of infection, loosening of the metal ware, or unexpected symptomology) participants will commence treatment according to their randomisation group (RG or UC).

### Development of the rehabilitation programme

The rehabilitation programme described in this protocol was developed by the CI in collaboration with the department of Physiotherapy UCLH, the Complex Spine Surgical Team (NHNN), and the study supervisors. The limited published studies suggest a potential benefit with complex rehabilitation over conventional exercise therapies [21, 22]. It was felt patients undergoing LFS had multi-dimensional needs [25] in keeping with the biopsychocosocial model of back pain [27]. This study will go some way to advancing the understanding of the needs of this patient group highlighted as urgent in a recent systematic review [24]. The rehabilitation protocol described is designed to optimise recovery through the provision of individualised, progressive exercise, education and peer support, employing principles of cognitive behavioural therapy (CBT) to help overcome maladaptive health beliefs.

In planning the current feasibility study a small-scale local exploratory study [26] was conducted. Compliance, willingness to participate and clinical outcomes were good which warranted the further evaluation described in this protocol.

### Rehabilitation group content

RG consists of 10 consecutive weekly outpatient appointments (maximum 90 min), including structured advice, progressive exercise and peer support. The delivery of the RG will employ principles of CBT, include a maximum of eight participants and be run in the physiotherapy gymnasium at UCLH, supervised by a senior physiotherapist with more than 10 years of experience of this subject group. All staff providing the RG will receive a minimum of 3 h of training in the delivery of the intervention.

**Fig. 2:**
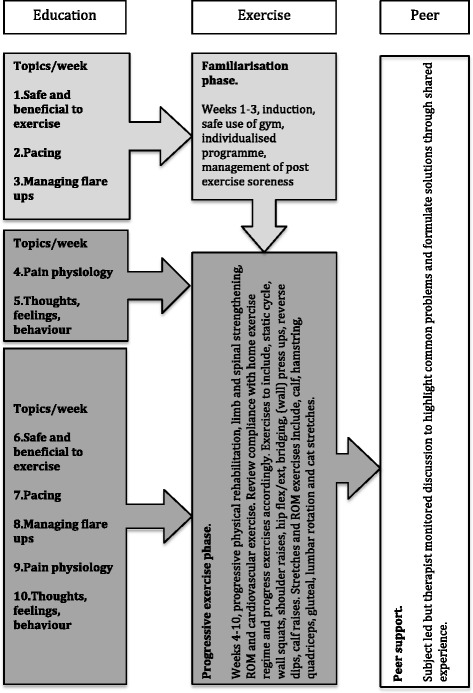
Rehabilitation protocol overview

The overarching aim of the RG is to provide clear, consistent educational messages, progressive physical rehabilitation (including home exercise) using low-tech exercises not requiring complex equipment or supervision with peer support. Each RG will commence with an initial, brief education session as outlined in Fig. 2. This is followed by an individualised, progressive exercise regime supervised by the therapist concluding with a monitored peer support session.

#### Education component

This comprises of five brief pre-planned educational sessions (maximum 20 min duration), conveying clear and concise principles relating to improving physical function and managing pain, beginning with topic 1 and progressing to topic 5. This includes topics such as the benefits of exercise, pacing, pain mechanisms, hurt≠harm and the over-/under-activity cycle to correct maladaptive beliefs and thus facilitate recovery, possibly via cognitive restructuring and *in vivo* exposure learning [28–30]. Topics will be briefly explored within the personal experience of the participants and open discussions will be encouraged. Each session will commence by revisiting the topic(s) from the previous week. In this way the educational component builds as participants recover lost function. After the first 5 weeks each topic is revisited to help participants apply the early-learned principles into their functional recovery.

#### Exercise component

This comprises an individualised, progressive, physical rehabilitation programme with exercises aimed at improving cardiovascular function, limb and spinal strength and flexibility. Assistance will be given as required. The selected exercises are intentionally low-tech and do not require complex equipment or supervision; as such they can easily be replicated outside the hospital. Records of training volume/intensity will be kept. Weeks 1 to 3 comprise the familiarisation phase allowing participants to become acquainted with the gym environment and post exercise symptom response. Following this the exercise load will be progressed according to individual ability. Exercise diaries to record activity between groups, problems or post exercise symptom change will be provided and reviewed weekly to monitor progress and compliance for reporting.

#### Peer support component

A peer-led discussion will be held at the end of each session (maximum 20 min duration) during which participants will be encouraged to discuss common problems and work towards identifying solutions based on shared experience. The physiotherapist will monitor this discussion to ensure no reinforcement of maladaptive beliefs and identify any psychosocial blocks to recovery.

### Usual care arm

This will include the same postoperative advice that all participants receive in that they should steadily, self-progress their walking mobility up to a maximum of 2 miles per day. The provision of subsequent physiotherapy, analgesia or pain management services will be entirely at the discretion of the surgical team or GP. Participation in this study will not preclude the provision of any rehabilitation that is deemed necessary; rehabilitation requirement will be recorded and reported.

### Primary and secondary outcome measures

Primary outcome measures include a range of markers evaluating the feasibility of the protocol, specifically the recruitment process, compliance and acceptability, as follows. For the recruitment process these will be the numbers of: eligible patients; patients who accept the PIS (following phone contact from the CI); patients willing to discuss trial participation (with the CI at PAS), and participants who provide consent and BLD. For compliance these will be: the rehabilitation intervention (review of exercise diaries and attendance); usual care (attendance at other rehabilitation settings/type of rehabilitation provided); the trial protocol, and the rate of/reasons for attrition. For the acceptability of the rehabilitation group and UC this will be qualitative evaluation to better understand participants’ perceptions of surgical after care in RG and UC.

The secondary evaluation utilises a cluster of quantitative outcome measures. The differences between groups will be investigated taking into account the variation between patients and the longitudinal nature of the data, using such methods as repeated measures analysis of variance (ANOVA) or multilevel regression as appropriate. The longitudinal approach will allow us to use all observations even if a patient is lost to follow up. The observed effect size and inter-patient variance will allow us to propose a suitable sample size for future research. The measures include: the Oswestry disability index (ODI), which measures self-reported level of disability [31]; the aggregated functional performance test (AFPT), which is the aggregated time (in seconds) for the subject to sit-to-stand, walk 50 yards, and make a stair ascent/descent, to quantify the level of physical function [32, 33]; the pain self-efficacy questionnaire (PSEQ), which measures self-reported ability to self-manage pain [34]; the hospital anxiety and depression scale (HADS), a measure of hospital-related level of anxiety and depression [35]; the European quality of life-5 dimensions (EQ-5D) measure of health-related quality of life across a range of indicators [29], and the client services receipt inventory (CSRI), an economic questionnaire customised to the patients’ needs post LFS [36].

### Data collection

Anthropometric data, including age, sex, height, body weight, comorbidities and relevant medical history of back problem (diagnosis, duration, previous conservative/surgical management, et cetera) and socioeconomic status (work status, sick leave, et cetera), will be recorded at baseline. All quantitative measures will be recorded at baseline (pre-operatively), and at 3, 6 and 12 months postoperatively. Data will be stored on secure hospital-based, password-protected computers. Each participant will have unique alpha-numeric codes assigned (the CI and the primary supervisor having access). Double data-entry and random regular third-party checks will take place to ensure accuracy.

### Qualitative analysis

A nested qualitative study will evaluate participants’ experiences. Six months after LFS participants will be asked to complete a short commentary detailing their experiences of the postoperative period. The content of the commentaries will not be analysed exhaustively, but be used to describe participants’ experiences of their postoperative management, its acceptability, facilitators and barriers, how to improve the RG/UC, and to identify 10 participants from each group to undertake semi-structured interviews and to inform the content of these interviews.

Semi-structured interviews will be conducted (maximum 1 h) from a purposive sample (n = 20; RG = 10, UC = 10) chosen from their commentaries to reflect the extreme and the midpoint opinions. The interviews will probe participant experience to achieve a deeper understanding of the perceptions and feelings related to LFS after care. Interviews will be recorded, transcribed and analysed. Commentary data and audio-recordings of the interviews will be anonymised, assigned pseudonyms and imported into a qualitative data analysis package (NVIVO). The interviews and commentary data will be analysed thematically using an inductive and deductive approach, to ensure the full range of responses are represented. Codes generated from the data will be assigned to portions of the text, a portion of transcripts independently double-coded by MH/FJ, ambiguities or differences will be discussed and resolved, ensuring no important issues are overlooked and an accurate, clear and balanced interpretation of the data is achieved [37–40]. As subsequent interviews are analysed, codes will be developed iteratively. Codes will be grouped into themes to develop outputs that identify key areas of value or challenges in both RG and UC. If participants are willing, brief, limited telephone interviews with participants who withdraw from either group will be performed to endeavor to establish the reasons for non-compliance/attendance.

## Discussion

It is anticipated that this study will evaluate the feasibility of providing complex, structured, progressive rehabilitation 3 months following technically successful LFS. We anticipate demonstrating that this is feasible. We expect participants to be compliant with the rehabilitation intervention. We also expect the qualitative analysis to demonstrate satisfaction amongst participants in the RG. This trial is not designed to demonstrate clinical efficacy, however, we expect evidence of reduced disability (ODI) amongst participants receiving RG compared with UC. We will report on the characteristics (including population mean, SD and effect size) to help achieve a detailed analysis of the patient group and thus a robust framework for the development of future studies evaluating clinical efficacy.

We aim to achieve a better understanding of the rehabilitative requirements of participants following LFS and a mechanism by which rehabilitation to address those needs may be robustly analysed, allowing us to shape future trials to optimise recovery. A dissemination plan including publication in open access peer-reviewed journals following the CONSORT principles [41] and conference presentations is in place.

## Trial status

Currently the trial has received ethical approval and is recruiting the first subjects.

## Additional files

Additional file 1:
**Patient information sheet: sheet given to patients prior to seeking consent.**
Additional file 2:
**Consent form: form for the recording of informed, written consent from participants.**

## Abbreviations

AFPT: aggregated functional performance test
ANOVA: analysis of variance
BLD: baseline date
CBT: cognitive behavioural therapy
CI: Chief Investigator
CLBP: chronic low back pain
CSRI: client services receipt inventory
EQ-5D: European quality of life-5 dimensions
GP: general practitioner
HADS: hospital anxiety and depression index
HEI: higher educational institute
LFS: lumbar fusion surgery
NHNN: National Hospital for Neurology and Neurosurgery
NHS: National Health Service
PIS: patient information sheet
PSEQ: pain self-efficacy questionnaire
REC: Research Ethics Committee
REFS: rehabilitation following fusion study
RG: rehabilitation group
ODI: Oswestry disability index
PAS: pre-operative assessment
UC: usual care
UCLH: University College London Hospital
YLD: years lived with disability
