# Supplementary material for: Evaluating rehabilitation following lumbar fusion surgery (REFS): study protocol for a randomised controlled trial
Source: Trials. 2015 Jun 4;16:251. doi: 10.1186/s13063-015-0751-9 (PMC4469118; doi:10.1186/s13063-015-0751-9)
Supplement: Additional file 1: — Patient information sheet: sheet given to patients prior to seeking consent. [file 13063_2015_751_MOESM1_ESM.docx]

**(Additional file 2)**

**Patient information sheet**

**Study title; Evaluating a rehabilitation protocol following lumbar fusion surgery.**

**Version 2, 02/06/2014**

**Invitation to participate;**

We would like to invite you to take part in our research study. Before you decide we would like you to understand why the research is being done and what it would involve for you. **One of our team will go through the information sheet with you and answer any questions you have.** We’d suggest this should take about 10 minutes.

You should feel free to talk to friends, family or the research team about the study if you wish.

(Part 1 tells you the purpose of this study and what will happen to you if you take part. Part 2 gives you more detailed information about the conduct of the study).

Please ask us if there is anything that is not clear. You should feel free to call or email us at the address below if there is anything that you would like to discuss further at any stage.

**What is the purpose of the study?**

This study is being carried out to see if it is possible to provide group rehabilitation 3 months after lumbar fusion surgery.

At the moment less than half the people having lower back fusion surgery have physiotherapy after the operation. The majority of patients do not have any formal rehabilitation at all.

Our experience and other research centers have shown that rehabilitation can improve the recovery from this lower back (lumbar) fusion surgery.

**Why have I been invited?**

You have been invited to participate in this study because you are planning on having lumbar fusion surgery. When we spoke you expressed an interest in being involved and we hope you will consider taking part.

**Do I have to take part?**

No. It is up to you to decide to join the study. We will describe the study to you and go through this information sheet with you. We very much want you to have all the information you need to make an informed decision. Please ask us if there is anything that is not clear to you.

You are free to withdraw at any time, without giving a reason. If you do decide to leave the study any data that you have provided will be kept and used in the results of the study. This will not affect the standard of care you receive.

**What will happen to me if I take part?**

Before you agree to take part we will ask you to read this sheet and ask any questions that you may have about the study. We would like to you to fully understand the process and have an opportunity to discuss this with your friends and family. If you still feel as though you would like to be part of the study we will ask you to sign a consent form and we will let your GP know. We will then ask you to fill out some questionnaires and do a very short test of you physical function. This will be done by the research team and will take approximately 15 minutes.

We will also allocate you to either the rehabilitation group (RG) or the usual care group (UC). The decision as to which group you are allocated is completely random, similar to tossing a coin. Neither you, the research team or your consultant will have any control over which group you are allocated to. This is so we get a fair understanding of how people respond to each group. Therefore half the people in this study will have formal rehabilitation at UCLH in the physiotherapy department and half will have usual care.

After you have agreed to join the study and been allocated to one of the two groups you will then have your lumbar fusion operation as planned. If you decide not to take part in the study your surgery will still go ahead completely as planned. After the surgery you will be limited in what you can do for 3 months and after this you will have another check up appointment with your surgical team. This is normal clinical practice and will be the same for people in the study as well as those who are not. If the surgeon is happy with your progress at 3 months after the fusion operation you will be able to begin either your rehabilitation or usual care depending on which group you are allocated to.

Those who are asked to attend the rehabilitation group will be expected to go to UCLH physiotherapy department every week for 10 weeks. Each session will last no more than 90 minutes and will consist of supervised exercises, education and peer support. These groups will be run by a senior and experienced physiotherapist. At the end of the 10 weeks there will be no more treatment and your surgical team will see you in clinic to assess your progress. They will continue to provide your clinical care and will be able to help with any concerns you may have.

Those people who are not allocated to the rehabilitation group will have the current usual care. This consists of advice to keep active and gradually improve your outdoor walking, you or your surgeon may feel that you need some extra help and you may be referred to physiotherapy. You will be able to access any care that your surgeon feels necessary (eg; physiotherapy) but you will not be referred to the rehabilitation group.

Everyone in the study will also be asked to fill in some short questionnaires and repeat the physical function test at 3, 6 and 12 months after the surgery. This will not take more than about 15 minutes on each occasion. You will also be asked to consider undertaking an interview to better understand your opinions about the surgical after care you received.

The flow chart below shows how the study will progress, please feel free to discuss this with a member of the research team if you have any concerns or questions.

**Trial Flow Chart General Overview**

Patient consent and randomisation

Satisfactory 3 month post operative review with surgical team and questionnaires

Sample identified and formal interviews

Usual care

Rehabilitation group 10 weeks

Usual hospital and post operative care for 3 months

Surgical fusion

Questionnaires and physical test

Not fit to continue study

6 month review with surgical team and questionnaires including brief interview

12 month review with surgical team and questionnaires

Study ends participants have ongoing care with surgical team

**Expenses**

Those people who are asked to attend the rehabilitation group will have a one day zone 1-6 travel card provided to help meet the cost of attending the group.

**What will I have to do?**

Those people who are randomised to the rehabilitation group will be asked to attend the rehabilitation group. This group will run for 10 weeks and each session lasts a maximum of 90 minutes. It involves advice and exercise in a small group (maximum of 8 people) of people that have had the same operation. These classes will be run at fixed times at UCLH and we will provide a choice of appointment times to try and make this as convenient as possible.

Some people may not be able to attend all the appointments. This is expected but we do anticipate that people will attend at least half of the available rehabilitation sessions.

Those who are allocated to receive usual care will not have to do anything that they would not normally do after this operation. If they would like to have treatment with a physiotherapist then their surgical team or GP will be able to request this.

Everyone that agrees to take part will be asked to fill out a few short questionnaires. These are very similar to those which are normally completed as part of the surgery but will take a few minutes more. Each time you complete the questionnaires (4 times in total) it will take approximately 15 minutes.

**What are the alternative treatments?**

After this lower back fusion operation some people have physiotherapy. It is not clear how often this is required but less than half the people that have lumbar fusion surgery will have some physiotherapy. This can be arranged for you by your surgical team if it is thought necessary.

**What are the possible disadvantages/risks of taking part?**

If you are allocated to the rehabilitation group we will be asking you to spend time and effort attending the rehabilitation classes and doing some exercise at home.

You may feel that the study did not reflect your concerns after the fusion surgery. We will be collecting a short summary from participants about what they thought was good or bad about the care they received after the surgery. This would be an opportunity for you to let us know what you thought about the care you received.

It may be that people in the rehabilitation group are happier with their treatment than those in the usual care group. If this is the case we would offer rehabilitation to all patients at the end of the study.

**What are the possible benefits of taking part?**

We cannot promise the study will help you but our work has suggested that this type of rehabilitation will help improve your physical well being after the surgery. We hope this will help you do more, get fitter and reduce pain. The information we get from this study will help improve the treatment of people following lower back fusion surgery. Other studies have suggested that this approach should be beneficial.

**What happens when the research stops?**

When the research stops we will publish our findings in journals and at conferences. You will never be named in any of these publications and all data will be treated with the utmost confidentiality.

We hope that those people who have been able to increase their exercise will be able to continue this but no formal arrangements to do this under the NHS will be made.

**Part 2 of the patient information sheet.**

**What if new relevant information becomes available?**

Sometimes we get new information about the treatment being studied. If this happens, your researcher will tell you and discuss whether you should continue in the study. If you decide not to carry on, your researcher will make arrangements for your care to continue. If you decide to continue in the study they may ask you to sign an agreement outlining the discussion.

OR

If this happens, your researcher might consider you should withdraw from the study. They will explain the reasons and arrange for your care to continue.

OR

If the study is stopped for any other reason, we will tell you and arrange your continuing care.

**What will happen if I don’t want to carry on with the study?**

You will be free to withdraw from the study at anytime without giving any reason. Any data that you have provided up to the point that you withdraw would remain in the study. This may be used for evaluation at a future date.

**What if there is a problem?**

If there is a problem there are compensation schemes in place to look after you. The first point of contact should be the Chief Investigator (CI) who has contact details at the end of this information sheet. If however you would rather contact PALS (patient advice and liason service) they will be able to advise you, details are at the end of the sheet.

**Will my taking part in this study be kept confidential?**

Yes all of your details will be confidential and all your information will be stored under a code rather than your name. This will ensure that all data is anonymous, only members of the research team will have access to it. It will be collected by the named CI only and they have a contract of employment with the NHS Trust. It will only be stored on NHS Trust computers and these will all be password protected and stored in secure clinical areas. All data will be kept for 5 years after the conclusion of the study. Personal data will be stored under a code rather than your name and will only be kept for 3 months after the end of the study. All audio recordings will be kept for 1 month after the final audio data has been analysed at the end of the study. After this time they will be destroyed confidentially.

**Will my GP be informed?**

Yes. Your GP will be informed about your participation in the study.

**What will happen to the results of the research study?**

The results of the study will be published in journals and at conferences. This will always be done without any direct reference to you as an individual. This may take 5 years from the beginning of the study. A lay summary document will be provided for interested participants.

**Who is organizing and funding this study?**

This study is being funded by the National Institute for Health Research (NIHR) this is as part of a research fellowship. Mr Jim Greenwood is organising this study in collaboration with University College London Hospital (UCLH). The study is sponsored by the Research and Development department of UCLH NHS Foundation Trust.

**Who has reviewed this study?**

All research in the NHS is looked at by an independent group of people called a Research Ethics Committee. This study has been reviewed and given a favorable opinion by REC Queen Square, number 14/LO/0748.

**Further information and contact details.**

The main person to contact regarding this study is Mr Jim Greenwood, his contact details are at the top of this information sheet. He is the chief investigator, (CI).

Chief Investigator, Jim Greenwood,

email; [james.greenwood@uclh.nhs.uk](mailto:james.greenwood@uclh.nhs.uk),

phone; 02034483568

If you remain unhappy and wish to complain formally you can do this through the NHS complaints procedure. Via the link below.

[www.nhs.uk/choiceinthenhs/rightsandpledges/complaints/Pages/AboutNHScompliants.aspx](http://www.nhs.uk/choiceinthenhs/rightsandpledges/complaints/Pages/AboutNHScompliants.aspx)

Details can be obtained through your local Patient and Advice and Liason Service (PALS),

PALS,

University College Hospital,

Elizabeth Garrett Anderson Wing,

235 Euston Rd,

London,

NW12BU.

Tel; 02063809975

Administration Support,

Mrs Pauline Hutchinson-Haynes,

Internal Box 6,

Victor Horsely Dept of Neurosurgery,

NHNN,

Queen Square,

London,

WC13BG.

Tel; 02034483568
