# Supplementary material for: Evaluating rehabilitation following lumbar fusion surgery (REFS): study protocol for a randomised controlled trial
Source: Trials. 2015 Jun 4;16:251. doi: 10.1186/s13063-015-0751-9 (PMC4469118; doi:10.1186/s13063-015-0751-9)
Supplement: Additional file 2: — Consent form: form for the recording of informed, written consent from participants. [file 13063_2015_751_MOESM2_ESM.docx]

**(Additional file 2 Consent form)**

Centre Number:

Study Number: 14/LO/0748

Patient Identification Number for this trial:

**CONSENT FORM**

**Title of Project: Evaluating a rehabilitation protocol following lumbar fusion surgery. A feasibility trial.**

Name of Researcher: **Jim Greenwood**

(Please initial box)

1. I confirm that I have read and understand the information sheet dated 02/06/2014 (version 2) for the above study. I have had the opportunity to consider the information, ask questions and have had these answered satisfactorily.

2. I understand that my participation is voluntary and that I am free to withdraw at any time without giving any reason, without my medical care or legal rights being affected.

3. I understand that relevant sections of my medical notes and data collected during the study (anonymised), may be looked at by individuals from, from regulatory authorities or from the NHS Trust, where it is relevant to my taking part in this research. I give permission for these individuals to have access to my records and for the inclusion of this data in future studies.

4. I agree to the use of my voice recording.

5. I agree to my GP being informed of my participation in the study.

6. I agree to take part in the above study.

------------------------------------------- ---------------------------------------- ------------------------------------

Name of Patient Date Signature

------------------------------------------- ----------------------------------------- ------------------------------------

Name of Person Date Signature

taking consent

When completed:

1 for participant;

1 for researcher site file;

1 (original) to be kept in medical notes.
